# Adoptive Cell Therapy in Pediatric and Young Adult Solid Tumors: Current Status and Future Directions

**DOI:** 10.3389/fimmu.2022.846346

**Published:** 2022-02-22

**Authors:** John A. Ligon, Kristin M. Wessel, Nirali N. Shah, John Glod

**Affiliations:** ^1^ Pediatric Oncology Branch, Center for Cancer Research, National Cancer Institute, National Institutes of Health, Bethesda, MD, United States; ^2^ Department of Pediatrics, Division of Hematology/Oncology, University of Florida College of Medicine, Gainesville, FL, United States

**Keywords:** adoptive cell immunotherapy, solid tumor, tumor microenvironment, immune evasion, CAR (chimeric antigen receptor)

## Abstract

Advances from novel adoptive cellular therapies have yet to be fully realized for the treatment of children and young adults with solid tumors. This review discusses the strategies and preliminary results, including T-cell, NK-cell and myeloid cell-based therapies. While each of these approaches have shown some early promise, there remain challenges. These include poor trafficking to the tumor as well as a hostile tumor microenvironment with numerous immunosuppressive mechanisms which result in exhaustion of cellular therapies. We then turn our attention to new strategies proposed to address these challenges including novel clinical trials that are ongoing and in development.

## Introduction

Immunologically “hot” solid tumors (e.g. melanoma) (1) with a tumor microenvironment (TME) marked by infiltrating CD8+ T-cells (2, 3), high programmed death ligand 1 (PD-L1) expression (4), or a high tumor mutational burden have shown remarkable responses to immunotherapy including immune checkpoint inhibitors (ICIs) (5). Unfortunately, these benefits have not extended to “cold” tumors (e.g. prostate or pancreatic cancer) (1) where T-cells are either entirely absent (“immune desert”) or sequestered at the periphery (“immune-excluded”) (3, 6). Many pediatric/adolescent and young adult solid tumors are cold tumors (7, 8) and have failed to respond to ICIs (9).

**Figure 1:**
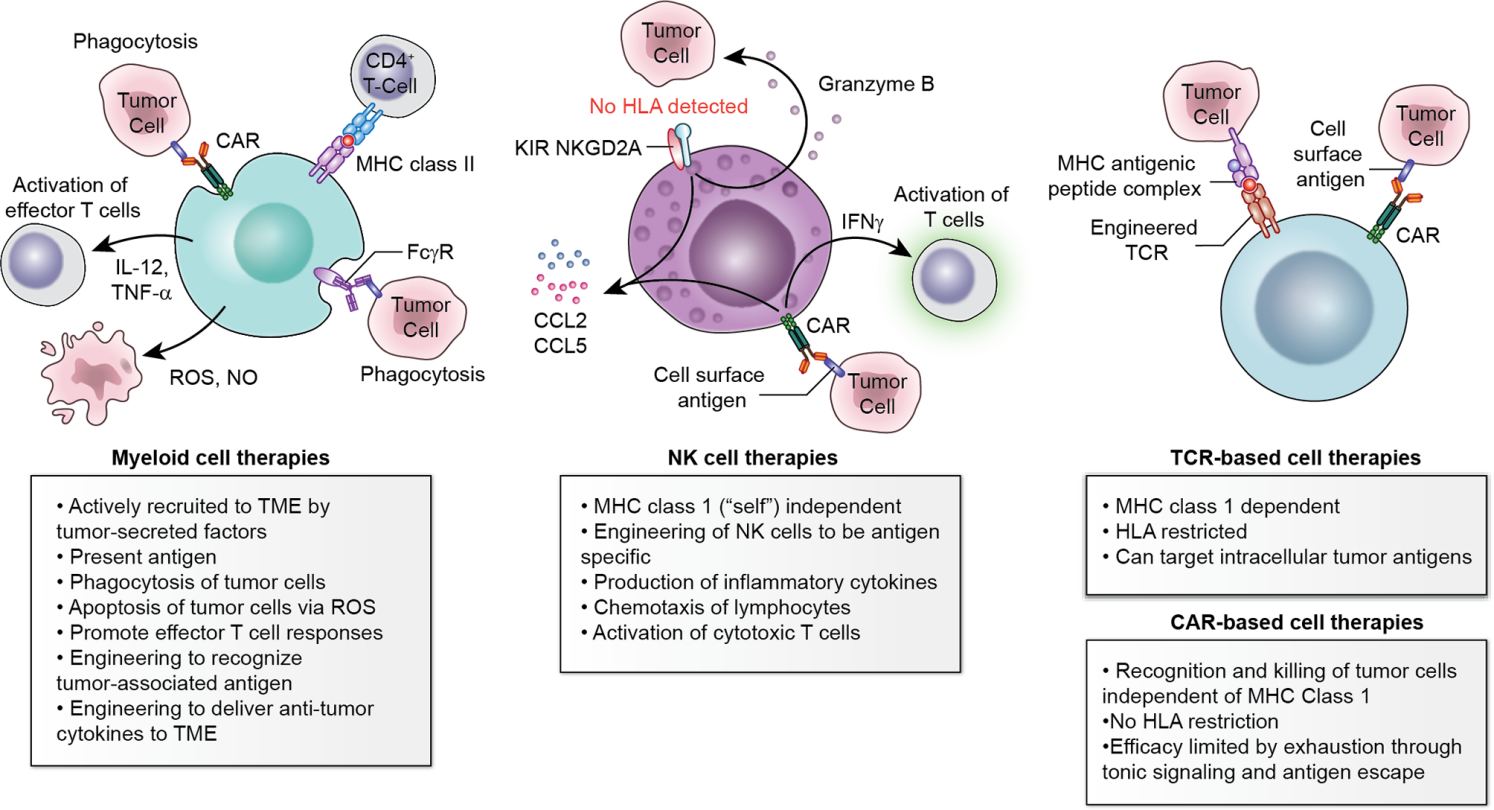
Multiple cell types available to engineer for adoptive cellular therapy. Myeloid cells, NK cells, and T-cell-based therapies each have advantages and disadvantages which should be considered within the context of the histology to be targeted.

Several approaches have attempted to harness cellular therapy to cure these tumors. Autologous hematopoietic stem cell transplant (HSCT) has enabled maximal chemotherapy dosing in susceptible tumors with varying levels of effectiveness in neuroblastoma (10), Ewing sarcoma (11), breast cancer (12), retinoblastoma (13), hepatoblastoma (14), and other diseases. Recently some groups have piloted allogeneic HSCT to treat solid tumors. Though durable responses are rare, evidence for graft-vs-tumor effect has been observed (15). Finally, as adoptive cellular therapy (ACT) has proven transformative for leukemia and lymphoma, the development of novel ACT for solid tumors has exploded (**Figure 1**). In this review, we discuss ACT in solid tumors in clinical development, consider challenges plaguing the field, and highlight proposed strategies which will be tested in future clinical trials.

## T-Cell Based Therapies

T-cells are critical in immune surveillance for cancer. The T-cell receptor (TCR) can recognize cancer-specific antigens processed by major histocompatibility complex (MHC) and presented on the cell surface. TCR engagement by MHC-presented non-self antigens leads to activation and T-cell mediated killing (16). T-cell cytotoxicity in solid tumors has been leveraged using both native T-cells and autologous T-cells genetically engineered to express a specific TCR. Future efforts in allogeneic “off-the-shelf” approaches are being actively studied.

### Tumor Infiltrating Lymphocytes (TILs)

Early studies demonstrated that heterogeneous tumor infiltrating lymphocytes (TILs) collected from a freshly-resected tumor and expanded *in vitro* were able to specifically lyse autologous tumor (17). Subsequent clinical investigations showed transient responses in patients with metastatic melanoma after TIL infusion, typically under high IL-2 conditions (17). Early TIL trials reported responses in 49-72% of patients with melanoma (18, 19). Pretreatment with lymphodepleting chemotherapy led to improved TIL persistence (18) and recent advances include selection of TILs that recognize patient-specific tumor antigens using single cell sequencing (20). Selected autologous TILs have shown activity in several epithelial malignancies (21, 22).

While advances using TILs continue, the inability to isolate and effectively expand TILs from some solid tumors remains a challenge.

### Engineered TCR-Based ACT

Initial attempts at engineering T-cells for ACT concentrated on genetic engineering of specific TCRs into autologous T-cells collected *via* peripheral blood apheresis with subsequent reinfusion.

Expression of cancer/testis antigens (CTAs) including melanoma antigen gene (MAGE) family proteins, synovial sarcoma X breakpoint (SSX) family proteins, and New York esophageal squamous cell carcinoma (NY-ESO-1) is normally restricted to the germline. However, solid tumors including melanoma, SS, myxoid/round cell liposarcoma (MRCL), and osteosarcoma express CTAs. Robbins and colleagues targeted NY-ESO-1 using a transduced TCR recognizing the peptide epitope SLLMWITQC in the context of HLA-A*02. Transgenic T-cells combined with IL-2 following lymphodepletion led to responses in 5/11 patients with SS and 2/11 patients with melanoma (23). A subsequent study showed responses in 6/12 patients with NY-ESO-1+ SS in an initial cohort (24), with one complete response (CR) and 14 partial responses (PR) in the first 42 patients (25). This response rate represents a potentially significant improvement over previous therapies for SS (26). These T-cells maintained clonal diversity over time and persisting cells were primarily of central memory and stem cell memory populations (24). Ongoing trials are further investigating genetically engineered NY-ESO-1 targeting autologous T-cells in solid tumors including SS, MRCL and non-small cell lung cancer (NCT02992743, NCT03967223, NCT03709706).

Other successfully targeted CTAs include MAGE-A3 and MAGE-A4. Seventeen patients were treated in a dose escalation study of autologous T-cells genetically modified to express an MHC class II-restricted TCR recognizing MAGE-A3 combined with IL-2 (27). One patient with cervical cancer had a CR and several PRs were observed in patients with esophageal cancer, urothelial cancer, and osteosarcoma. Despite encouraging responses, the significant neurotoxicity observed in this and a subsequent trial targeting MAGE-A3 has hampered development of this strategy (28). In a phase I trial of a TCR developed in a transgenic murine model and recognizing residues 112-120 (KVAELVHFL) of MAGE-A3, 3/9 patients developed significant neurotoxicity (29). Preliminary data for the SURPASS trial utilizing autologous T-cells transduced with a MAGE-A4 TCR and CD8a co-receptor reported 2/5 patients with PRs (30). A MAGE-A4 targeting TCR is also being evaluated in a phase II study for patients with SS and MRCL (NCT04044768). Additional TCR-based strategies targeting CTAs are in development (31).

Viral antigens have also been successfully targeted for treating solid tumors using ACT. TILs targeting human papillomavirus (HPV) antigens E6 and E7 have shown efficacy in early phase clinical trials in HPV-associated carcinomas with responses in 5/18 patients with cervical cancer and 2/11 with head and neck cancer (32). Subsequent work identified TCRs recognizing epitopes of HPV16 E6 and E7 in the context of HLA-A*02:01 and T-cells genetically engineered to express these TCRs led to responses in two early phase studies (33, 34). Epstein-Barr virus (EBV) is associated with several solid tumors (e.g., nasopharyngeal carcinoma [NPC] and post-transplant lymphoproliferative disorder [PTLD]). EBV-specific cytotoxic T-lymphocytes (CTLs) were tested to treat PTLD following HSCT (35). EBV-specific CTLs resulted in PR for 2/10 patients with EBV-associated NPC (36). A phase 3 trial comparing chemotherapy with EBV-specific CTLs combined with chemotherapy for NPC is underway (NCT02578641). TCR-based therapy has also been explored for Merkel cell carcinoma, a skin cancer associated with Merkel cell polyomavirus (37). Autologous T-cells with TCRs recognizing an epitope of Merkel cell polyomavirus, large T antigen and small T antigen, led to durable regression of metastatic lesions in several patients (38).

### CART-Based ACT

HLA-restriction (limiting patient access) and reliance on tumor MHC expression have limited TCR-based therapy utility. Chimeric antigen receptor T-cells (CART) are autologous T-cells engineered *ex vivo* to enable MHC-independent tumor cell killing without HLA restriction. First-generation CAR have 3 components: a specific antibody-derived single chain variable fragment (scFv), a hinge/transmembrane domain, and a T-cell signaling (CD3ζ) domain. Second-generation CAR incorporate one additional co-stimulatory domain, while third-generation CAR incorporate 2 additional co-stimulatory domains. Fourth-generation CAR, also known as TRUCKs (T cells redirected for antigen unrestricted cytokine-initiated killing) include a CAR-inducible transgene product, often pro-inflammatory cytokines which may enhance CART cytotoxicity and activate other immune cells in an immunosuppressive TME (39).

Human epidermal growth factor receptor 2 (HER2) is expressed on several solid tumor types and has attracted interest as a CART target. A clinical trial utilizing 10^10^ of a third-generation CART incorporating a scFV derived from the humanized monoclonal antibody trasztuzumab following lymphodepletion for HER2+ solid tumors. A patient with metastatic colorectal cancer developed fatal respiratory failure 15 minutes after CART infusion. This was thought to be due to massive cytokine release upon recognition of HER2 at low levels on lung epithelium and prompted concerns about the safety of HER2-CART (40). A subsequent HER2-CART trial in HER2+ sarcomas instead utilized the FRP5 scFV, omitted lymphodepletion, and selected a lower starting dose of 10^4^/m^2^. There were no dose-limiting toxicities, but also no CART expansion. Doses greater than 10^6^/m^2^ were associated with greater persistence. 4/17 evaluable patients had stable disease and 1 patient had a PR after a second CART infusion (41). To improve CART expansion and persistence, an ongoing phase I HER2-CART trial (NCT00902044) has incorporated lymphodepletion and HER2-CART doses up to 10^8^/m^2^. Thus far two CRs have been reported (42, 43).

Clinical experience with CART targeting the diaganglioside GD2, which is highly expressed on osteosarcoma, neuroblastoma, and many central nervous system (CNS) tumors, also suggests that lymphodepletion and adequate cell dose are important for CART expansion and persistence. A phase 1 trial utilizing first-generation GD2-CART without lymphodepletion in neuroblastoma demonstrated safety and clinical activity with 3 CRs, but showed limited expansion and persistence (44, 45). A subsequent trial (NCT02107963) utilized a third-generation GD2-CART with lymphodepletion, and demonstrated good expansion (46). A phase I study of a third-generation GD2-CART with or without lymphodepletion in relapsed/refractory neuroblastoma showed increased CART expansion following lymphodepletion (47). A phase I trial utilizing escalating doses of a second-generation GD2-CART with lymphodepletion of varying intensity in relapsed/refractory neuroblastoma showed regression of soft tissue and bone marrow disease following CART doses of at least 10^8^/m^2^ (48). GD2-CART have shown promising clinical activity in a phase 1 trial in H3K27M+ diffuse midline gliomas, which are universally fatal malignancies (NCT04196413) (49). Based on preclinical data suggesting that incorporation of IL-15 into CART further enhances persistence and cytotoxicity (50, 51), ongoing trials are utilizing GD2-CART and GD2-CAR-NKT cells engineered to express IL-15 (NCT03721068, NCT03294954).

The checkpoint molecule B7-H3(CD276) is another CART target of interest given its high expression on multiple solid tumor types. Preclinical studies have demonstrated encouraging activity of B7-H3-CART in various xenograft models (52–54). Clinical trials utilizing B7-H3-CART are underway in pediatric and adult solid tumors (NCT04897321, NCT04483778, NCT04432649, NCT05211557, NCT04670068) and CNS tumors (NCT04185038, NCT04385173, NCT04077866).

### TRuC™-T-Cell Based ACT

TCR fusion constructs (TRuCs) also enable HLA-independent cell killing. In contrast to CART, which incorporate only the intracellular signaling domain of the CD3ζ chain, TRuCs involve fusion of the scFv to the N-terminus of any of the other five subunits in the TCR complex. TRuCs are incorporated into the TCR on translation, engage the TCR complex upon activation, and are efficacious in solid tumor xenograft models (55). Anti-mesothelin TRuCs are being studied in a phase 1/2 clinical trial (NCT03907852) with preliminary evidence of activity, with 3/7 patients (2 with mesothelioma, 1 with ovarian cancer) achieving a PR (56).

## NK-Cells

NK-cells are innate immune cells and protect against infections and cancer (57, 58). Efforts to harness NK-cell biology for ACT in cancer treatment has gained considerable interest as an alternative to T-cell based immunotherapeutics. NK-cells possess qualities which may allow them to overcome the hostile TME (58–60). While T-cells recognize unique tumor antigens, NK-cell-mediated cytotoxicity depends on the sum of activating and inhibitory signals, including tumor cell lack of MHC class 1 expression or antibody-dependent cell-mediated cytotoxicity (61). Furthermore, NK-cells can produce inflammatory cytokines such as IFNγ and TNFα which can activate CD8+ TILs and enhance their cytotoxicity (62).

These properties allow NK-cells to be engineered or manipulated *via* different mechanisms from T-cell-centric immunotherapies. Examples include the administration of agonist cytokines or engineering NK-cells which constitutively secrete these cytokines (63). Others have proposed NK-cells which constitutively secrete chemotactic factors to recruit cytotoxic lymphocytes to the TME (64). Tri-specific NK-cell engagers (TriKEs) have been proposed to confer tumor-specificity to NK-cells and enhance NK-cell activation by engaging stimulatory receptors such as the IL-15 receptor (65, 66). Additionally, CAR NK-cells (CAR-NK) designed from stem cell progenitors represent another way to generate tumor-specific NK-cells. Attractively, CAR-NK may be less toxic and could be produced at lower cost than CART (67). Recent experience with CD19-CAR-NK in B-cell malignancies provides proof-of-concept that this strategy can be safely and effectively utilized and with potential for persistence (68). Barriers remain to production and monitoring of persistence of these cells, but additional alterations to the NK-cell product and manufacturing strategies have been proposed to mitigate these issues. Finally, NK-cells also express immune checkpoint molecules such as PD-1, and either combination with ICIs or intrinsic downregulation of these checkpoint molecules have been proposed as mechanisms to further enhance the efficacy of NK-cell-based approaches (69, 70).

## Myeloid Cell Therapies

Myeloid cells readily infiltrate primary tumors and metastases. Harnessing this property for ACT shows promise in the treatment of solid malignancies (71). Myeloid cells are highly plastic and may acquire a wide spectrum of immune-stimulatory or immune-suppressive phenotypes in response to the local milieu. Tumor associated macrophages (TAMs) are polarized to an anti-tumor M1 phenotype in response to pro-inflammatory factors such as IFNγ, GM-CSF and lipopolysaccharide. M1 TAMs promote Th1 responses, phagocytosis of tumor cells, and antigen presentation. Tumor-associated cytokines such as IL-10, IL-4, IL-13 and TGF-β promote polarization towards an immunosuppressive M2 phenotype. M2 TAMs promote tumor progression through mechanisms including angiogenesis, extracellular matrix (ECM) remodeling and regulatory T-cell recruitment (72). This M1/M2 classification is an oversimplification, however induction of an M1-like, anti-tumor phenotype is important for the success of myeloid-based ACT. The first myeloid-based ACT utilized macrophages polarized to the M1 phenotype *ex vivo* with IFNγ. Clinical trials showed limited efficacy, but these therapies were generally well-tolerated (73–75).

Subsequent work has focused on engineering myeloid cells towards a more potent and durable anti-tumor phenotype. Anti-HER2 CAR-macrophages (CARM) reduced tumor growth and prolonged survival while reprogramming the immune-suppressive TME in xenograft models (76). A first-in-human trial evaluating CARM is now underway in HER2-overexpressing solid tumors (NCT04660929). Preclinical work has shown that myeloid cells can also be used to deliver cargo to the TME. Administration of myeloid cells genetically engineered to express IL-12, a potent anti-tumor cytokine, resulted in durable cures in a syngeneic model of embryonal rhabdomyosarcoma through activation of T-cell responses in the tumor and metastatic microenvironment (77).

## Challenges in Solid Tumor ACT

**Figure 2:**
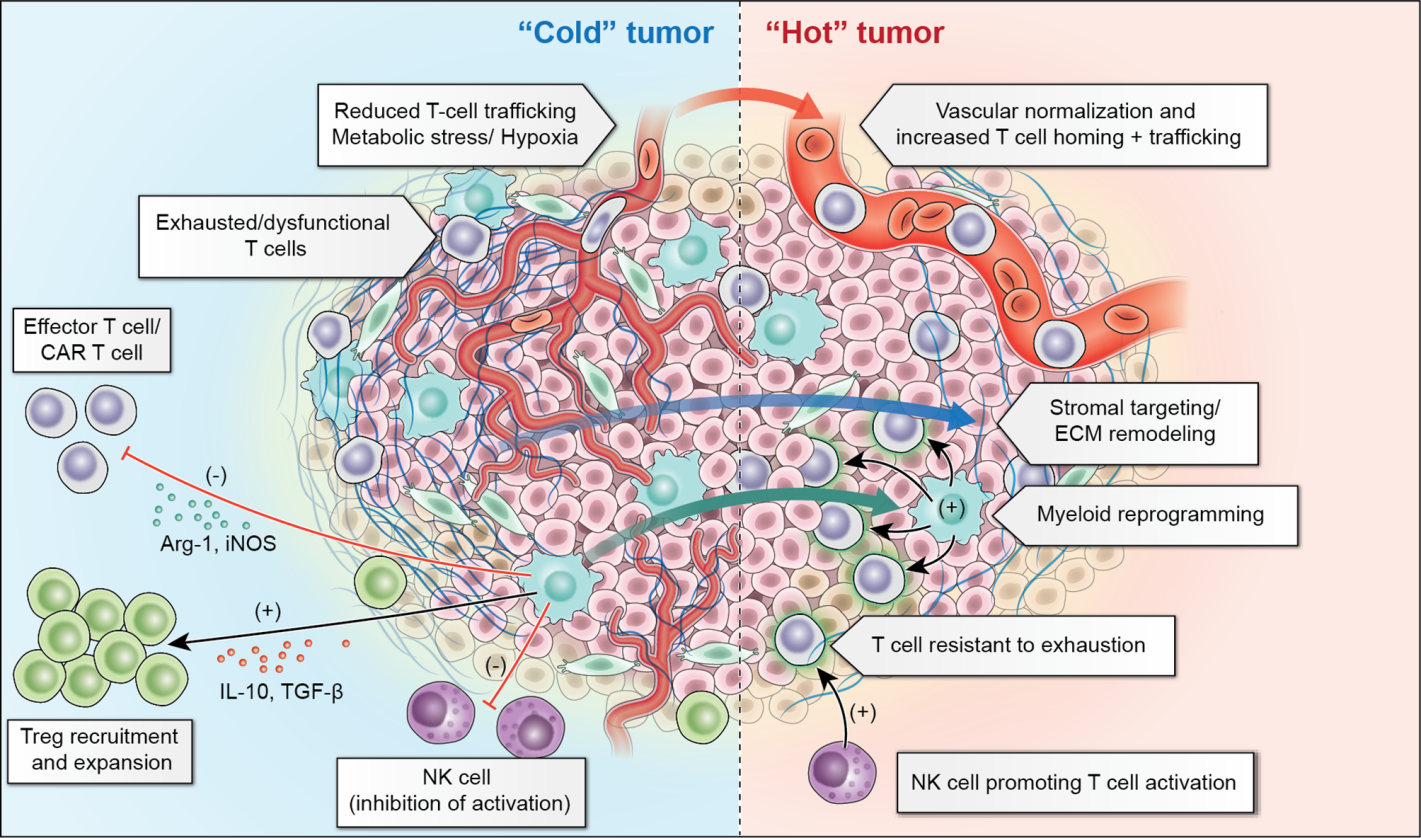
“Cold” solid tumors present a number of challenges within their tumor microenvironment including reduced trafficking related to abnormal tumor vasculature and resident inhibitory myeloid cells which recruit regulatory T cells (Treg) and lead to exhaustion of T-cells and NK-cells. Adoptive cellular therapies aim to overcome these challenges through vascular normalization and extracellular matrix (ECM) remodeling to promote improved trafficking, as well as myeloid cell reprogramming to diminish the inhibitory contribution of these cells. Additionally, T-cells which are resistant to inhibition or “armored” T-cells, or NK-cells which can augment T-cell responses may make it possible to overcome the inhibitory tumor microenvironment.

Significant remaining challenges for optimization of solid tumor ACT are outlined in this section. Additionally, we will summarize proposed strategies to overcome these challenges (**Figure 2**).

Selection of antigens such as GD2 (78) and CTAs (79), which are expressed on numerous solid tumors, leverages the possibility that a single ACT could be active across multiple histologies. However, few antigens are tumor-specific. Thus, identifying a target antigen which will allow tumor clearance without unacceptable normal tissue toxicity (on-target/off-tumor effect) is problematic. In addition to selecting the proper target antigen, low antigen density and antigen downregulation within heterogeneous solid TMEs have emerged as additional barriers to ACT (80, 81).

ACT trafficking is also challenging in solid tumors. Trafficking can be inhibited by physical barriers, loss of MHC class 1 expression, repellent cytokine gradients, expression of inhibitory ligands such as PD-L1, and abnormal tumor vasculature (82). CNS tumors are further shielded by the blood-brain barrier (83). If ACTs cannot traffic to the tumor and engage their target antigen, they fail to be activated and expand, leading to rapid loss of ACT.

Finally, the TME present in many solid tumors is hostile to the ACT. Tumors recruit immunosuppressive TAMs and myeloid-derived suppressor cells (MDSCs) (84) which express inhibitory molecules such as PD-L1 (84), secrete inactivating cytokines such as IL-10 (85), and promote a hypoxic TME (86) which can thwart ACT cytotoxicity. These tumor-sustaining programs promote rapid and irreversible ACT exhaustion, inhibit expansion, and result in failure of tumor clearance. Further ACT engineering or combination with agents to allow ACTs to overcome these challenges, will be necessary for ACT optimization in solid tumors.

## Discussion: Overcoming the Immune-Suppressive TME in ACT for Solid Tumors

Aberrant tumor vasculature and ECM deposition impede ACT trafficking. Regional ACT administration is one strategy to overcome this hurdle. A recent phase I trial demonstrated that intrapleural administration of mesothelin-CART combined with pembrolizumab was safe and feasible, and showed potential efficacy with 2 patients demonstrating metabolic CR on PET scan (87). Intraventricular CART administration for both primary brain tumors and CNS metastases is also under evaluation in early-phase clinical trials and in preclinical models (88, 89) (NCT04196413). Additionally, methods to disrupt the blood-brain barrier to allow trafficking of ACT to CNS tumors, such as focused ultrasound (90) or other mechanical or pharmacological methods (91) have been piloted.

Further genetic modification of ACTs to overcome and leverage features of the hostile TME is currently being explored. Many solid tumor types recruit TAMs by producing chemokines such as CXCL8 and CXCL2. Preclinical data suggest that chemokine secretion can be leveraged to enhance CART trafficking by engineering CART to express chemokine receptors. For example, CXCR2-modified GPC3-CART had improved trafficking in a hepatocellular carcinoma model (92), while a CXCR1/2-modified CD70-CART enhanced CART trafficking and efficacy in murine GBM, ovarian cancer and pancreatic cancer models (93). Many groups have also sought to generate a more “fit” ACT through enhanced cytokine secretion [thoroughly reviewed by Bell and Gottschalk (94)]. Additional modifications include creation of ACT which is resistant to exhaustion [e.g. DNA methyltransferase 3 alpha knock-out (95) or PD-1 deletion (96)] or tuning ACT to be effective despite low antigen density [e.g. c-Jun overexpression (97, 98)]. These modifications of ACTs are now entering clinical trials (e.g. TGF-βR knockout CART NCT04976218).

Tumor-associated vasculature is characterized by pericyte loss, resulting in leakiness and adhesion molecule down-regulation impairing T-cell migration into the tumor (99). VEGF inhibitors, which promote vascular normalization, may enhance CD8+ T-cell infiltration into tumors (100). Anti-VEGF agents have shown synergy with ICIs in select solid malignancies, resulting in FDA approval of these combinations in hepatocellular carcinoma and renal cell carcinoma (101). Preclinical studies suggest that antiangiogenics can also improve ACT trafficking (102, 103). Combining ACTs with antiangiogenics warrants further study in clinical trials.

ECM-remodeling agents may enhance the ability of ACTs to infiltrate tumors. In gastric cancer models, hyaluronic acid reduced mesothelin-CART infiltration, however these CART had superior efficacy when combined with infusion of a secreted form of the human hyaluronidase PH20 (104). CART engineered to express heparinase, which degrades heparan sulfate proteoglycans, showed superior anti-tumor activity and were associated with increased T-cell infiltration in preclinical models (105).

The solid TME contributes to T-cell exhaustion *via* multiple mechanisms, including repeated TCR stimulation and metabolic stress, thereby reducing the ACT efficacy. Engineering CART to reduce tonic signaling through incorporation of the 4-1BB costimulatory domain vs CD28 costimulatory domain showed reduction in CART exhaustion and enhanced persistence and efficacy in preclinical studies (106). Induction of transient rest periods in CART, such as by dasatinib utilization, has shown exhaustion reversal and improved efficacy (107). A dasatinib-containing culture platform is being used to manufacture GD2-CART in ongoing clinical trials (NCT04539366, NCT04196413). CART combination with ICIs is also under evaluation in clinical trials (108).

The ability of myeloid cells to orchestrate immune responses in the TME makes them an attractive therapeutic target. Low-dose chemotherapy has shown reduction of tumor MDSCs (109–111). MDSC differentiation with ATRA reduced their immune-suppressive function and enhanced efficacy of GD2-CART in preclinical models (112). In a pilot trial studying ipilimumab vs ipilimumab combined with ATRA, patients receiving ATRA had fewer circulating MDSCs (113). Inhibiting myeloid cell trafficking through CSF1R inhibition is another potential avenue to reduce myeloid cell immune-suppression in the TME. CSF1R-targeting agents are generally well-tolerated in the clinic, and the multi-TKI CSF1R inhibitor Pexidartinib is FDA-approved to treat tenosynovial giant-cell tumor (114, 115). Clinical trials studying CSF1R inhibitors with ICIs are underway (NCT02777710, NCT02829723, NCT03502330, NCT04848116, NCT02526017).

## Conclusion

While ACT has yet to yield the transformative results in solid tumors that CART have shown for hematologic malignancies, evidence exists that some patients with solid tumors may respond to ACT. T-cells, NK-cells, and myeloid cells have each been engineered to target these tumors, and each have advantages and unique challenges. Further engineering ACTs to overcome tumor immune resistance mechanisms and better understanding how to combine with TME-modifying agents will be critical to expanding the number of patients with solid tumors who may derive therapeutic benefit.

## Author Contributions

JL, KW, and JG wrote the first version of the manuscript. NS provided critical feedback and additions. All authors contributed to the final version of the manuscript.

## Author Disclaimer

The content of this publication does not necessarily reflect the views of policies of the Department of Health and Human Services, nor does mention of trade names, commercial products, or organizations imply endorsement by the U.S. Government.

## Conflict of Interest

The authors declare that the research was conducted in the absence of any commercial or financial relationships that could be construed as a potential conflict of interest.

## Publisher’s Note

All claims expressed in this article are solely those of the authors and do not necessarily represent those of their affiliated organizations, or those of the publisher, the editors and the reviewers. Any product that may be evaluated in this article, or claim that may be made by its manufacturer, is not guaranteed or endorsed by the publisher.
